# Understanding the factors influencing nurses in managing patients with diabetic ketoacidosis in the emergency departments of referral hospitals in Dar es Salaam, Tanzania: A descriptive qualitative study

**DOI:** 10.1371/journal.pone.0310414

**Published:** 2024-11-12

**Authors:** Ipyana Mbaga Kajembula, Kornel Izidory Matheo, Peter Damian Simchimba, Elizabeth Habili Masasi, Victor Chikwala, Joel Seme Ambikile

**Affiliations:** 1 School of Nursing, Muhimbili University of Health and Allied Sciences, Dar es Salaam, Tanzania; 2 Emergency Medical Department, Mwananyamala Regional Referral Hospital, Dar es Salaam, Tanzania; 3 Department of Community Nursing, School of Nursing, Muhimbili University of Health and Allied Sciences, Dar es Salaam, Tanzania; 4 Department of Clinical Nursing, School of Nursing, Muhimbili University of Health and Allied Sciences, Dar es Salaam, Tanzania; Gachon University Gil Medical Center, REPUBLIC OF KOREA

## Abstract

**Background:**

The quality of nursing management of diabetic ketoacidosis (DKA) in emergency departments may be associated with either increased or decreased length of hospital stay. Despite that patient with DKA need intensive care unit (ICU) admission, effective initial nursing management of DKA patients at the emergency department is important. Regarding factors influencing the effective management when caring for patients with DKA, it is unknown how Tanzanian nurses perceive these issues. Therefore, this study was aimed to explore nurses’ perceived factors influencing nursing management of DKA patients at emergency medical department (EMD) of two selected referral hospitals in Dar es Salaam, Tanzania.

**Methodology:**

A descriptive qualitative study design was conducted to explore nurses’ perspectives on the factors influencing nursing management of DKA patients. A total of twelve purposively selected nurses participated in in-depth interviews. The interview guide written in Kiswahili was used to collect data. All interviews were audio recorded and transcribed verbatim, and transcripts analyzed using qualitative content analysis.

**Findings:**

Two main themes emerged from the study including facilitators of DKA nursing management and barriers to DKA nursing management. Facilitators of DKA management encompassed three categories including nurses’ knowledge of DKA, the availability of DKA management protocol, and nurses’ skillset to enhance DKA management. On the other hand, barriers to DKA management had eight sub-categories including limited training on DKA management, lack of autonomy, decisions disagreement, delayed electrolyte results, scarcity of medical resources, shortage of nursing staffs, logistics in emergency care, and lack of specific-nursing management guideline.

**Conclusion:**

This study highlights facilitators and barriers to DKA management and underscores the need for comprehensive strategies to overcome these barriers and consolidate the facilitators to improve nurses’ capacity in managing patients with DKA.

## 1. Introduction

Diabetic ketoacidosis (DKA) is a life-threatening emergency that usually affects people with type 1 diabetes mellitus, although it can also happen to those with type 2 diabetes mellitus [1]. In type 2 diabetes mellitus patients, the most prevalent triggering causes for DKA include infections, untreated or undiagnosed diabetes, and noncompliance with diabetes treatment regimens [2, 3]. Clinically, DKA is defined by a metabolic triad of acidemia, hyperglycemia, and ketonemia [4]. Therefore, DKA is diagnosed when there is ketones in urine, blood glucose levels greater than 11 mmol/L, capillary blood pH is less than 7.3, and capillary bicarbonate levels are less than 15 mmol/L, and there is usually some degree of mental impairment [5, 6].

The prevalence of DKA vary across countries ranging from 22% to 88% [7–12]. A study conducted in the United States indicated that the prevalence of DKA among patients with type 1 diabetes mellitus was 46% between 2017 and 2019, which is lower than in Ethiopia, where 78.7% of patients diagnosed with type 1 diabetes mellitus had diabetic ketoacidosis at the time of diagnosis [13, 14]. Additionally, in Malaysia and Ethiopia, the reported occurrence of DKA among adults with type 2 diabetes mellitus is 51.1% and 40%, respectively [15, 16]. Despite the limited evidence exists regarding acute mortality from diabetes in sub-Saharan Africa [17], in 2021, Tanzania had the highest diabetes prevalence in Africa, with 12.3% of adults aged 20–70 years diagnosed with diabetes [18]. Based on a single tertiary hospital setting in Tanzania, the reported death rate of 24.1% from DKA is higher than either Malaysia or Ethiopia (17.6% and 12%, respectively) among DKA patients hospitalized to the intensive care unit (ICU) [16, 19, 20]. There is a suggestion that undiagnosed or inadequately treated type 1 diabetes may be the underlying cause of this acute mortality, as it rapidly advances to DKA [19]. Furthermore, in resource-limited settings, literature points that cerebral edema, sepsis, shock, and renal failure are the leading causes of death in children with DKA, whereas in older patients, severe hypokalemia, adult respiratory distress syndrome, and comorbid conditions such as pneumonia, acute myocardial infarction, and sepsis are significant predictors of mortality among DKA patients [21–23].

Comprehensive approaches to the management of DKA patients have been recommended in a number of ways, with the main ones being the restoration of circulatory volume, the correction of electrolyte and acidity imbalances, the resolution of hyperglycemia, and the treatment of the underlying cause of DKA [4, 24, 25]. This include giving insulin therapy with regular insulin, replenishing potassium, which is frequently reduced in DKA cases, and replenishing fluids with 0.9% normal saline [22–24]. Research has shown that higher death rates are linked to health care providers’ inappropriate management of DKA [26–28].

Data from medical facilities indicate that DKA patients should be managed in the intensive care unit (ICU) [22]; yet, effective management must begin at the emergency department (EMD). Nurses, often get involved in the management of critically ill patients including those with DKA. There are number of identified factors that generally affect nursing care in the emergency department and ultimately leading to poor patients outcome. Study conducted by Wolf *et al*., found that nurses work in the emergency department perceived inadequate staffing level being unsafe as associated with missed or delayed care, failure to rescuer and readmission [29]. Additional, another challenges include lack of individualized care, workload, ineffective clinical management and deficiencies in the clinical performance among nurses [30, 31]. With regard to DKA management, research points to difficulties nurses working in the Emergency Department encounter in providing DKA care, including a lack of autonomy and a lack of understanding of DKA management [32, 33]. Additionally, nursing management of patients must be individualized care, it has been experienced differently among nurses working at the Emergency Department, where it is found to be difficult to provide person-centered care [34]. Likewise, management patients with DKA need nursing care that are not only person-centered but also Evidenced Based Care (EBC) through utilization of DKA management protocol to improve the quality of nursing care and ultimately patient outcome.

Furthermore, one of the competencies a nurse working in an emergency department must have is the ability to provide effective initial care to acute critical patients, as it is linked with preventing the worsening patient’s condition [35]. Despite the competences that emergency nurse must have, comprehensive and continuous educational among nurses working in the Emergency Department is vital as it is associated with improved nursing care and ultimately patients outcome [36]. Emergency services in Tanzania have improved; in 2021, emergency services were expanded to the district level 101 with new EMD and EMD beds increased to 795 [37]. Therefore, nurses and other healthcare workers need to be conversant with effective initial management of DKA at the Emergency Medical Department level.

While there is limited empirical evidence supporting the critical role of nurses in managing patients with DKA, numerous studies have focused on nurses’ knowledge and attitudes of DM and insulin therapy [38, 39]. Notably, there is a gap in contemporary literature addressing the influencing factors towards nursing management of DKA patients at the emergency department. This research gap served as a compelling justification for undertaking this study.

## 2. Material and methods

### 2.1 Study design

We conducted a descriptive qualitative study, to look into the nurses’ perspectives on the factors influence nursing management of DKA patients in the emergency department [40]. The design was considered relevant as it provides a broad insight and description of the phenomena under investigation in a naturalistic, realistic, and comprehensive view [41, 42]. Also, qualitative descriptive design is favorable when little is known about the phenomenon under study.

### 2.2 Study setting

The study was conducted in two selected Regional Referral Hospitals (RRHs) in Dar es Salaam, namely; Mwananyamala and Temeke. Both are public hospitals with Emergency departments which initiate management of DM patients with DKA complications and other emergency patients before admitting them to medical wards or surgical wards. According to Demographic and Population Census of 2022, Temeke Municipal Council and Kinondoni Municipal Council had a population of 1,346,674 and 982,328 respectively [43].

### 2.3 Study population

The study involved licensed nurses working in the Emergency Medical Department. The study’s inclusion criteria were nurses working in an emergency medical department, having a nursing diploma as a minimum level of education, having at least six months of work experience, and giving consent to participate. Nurses having a background in midwifery or psychiatric nursing were excluded, as were those on yearly leave, sick leave, or performing other official tasks during data collection.

### 2.4 Sampling methods and procedure

Purposive sampling method was employed to recruit study participants, allowing researchers to select participants with needed information regarding nurses’ perspectives on the factors influencing care of DKA patient at the emergency department [44]. Sample size was determined by the saturation of information, where Monique Hennink and Bonnie N. Kaiser described that 9–17 interviews can provide saturation of information in face to face interviews [45]. Also, we employed the code meaning approach, in which researchers first examined interviews and identified codes, then they determined whether any new codes were identified in the interviews that followed until no new codes were found. The list of nurses working in the emergency department was obtained from the emergency department nurse in charge, and the list was reviewed to identify those who met the inclusion criteria and asked for an interview.

### 2.5 Data collection

Data were collected between august 2023 and November 2023 by authors and one experienced research assistant in qualitative data collection. Before beginning an interview, all participants were asked to provide written informed consent, which was obtained after they were informed about the study’s objectives, information gathering procedures, participants’ right to withdraw from the study, participant information confidentiality, participation in audio recording, and participant information publication. Participants received 10,000 Tanzanian Shillings (Tshs) (approximately 5 USD) as compensation for their time and transport as they were interviewed after working hours.

A semi-structured interview guide was developed after the literature was carefully reviewed and used for conducting interviews. To elicit the nurses’ viewpoints, experiences, and factors impacting the effective management of DKA patient at the emergency department, a series of initial and follow-up questions were constructed.

Twelve [12] face to face in-depth interviews were conducted in a quiet room that provided participants’ privacy and comfort at the respective hospital buildings [46]. The interviews were conducted at a convenient time decided by participants. All interviews were digitally audio-recorded to capture participant responses, each lasting for 40 to 60 minutes. During the interviews, the research assistant took notes regarding participant’s non-verbal cues, followed by a review of the filed notes to improve the following interviews and to note the emerging findings.

### 2.6 Data analysis

Management and analysis of data was an iterative process. All the recorded interviews (N = 12) were first transcribed verbatim in Swahili and then translated into English [47]. The transcripts were manually analyzed using qualitative content analysis as highlighted by Graneheim and Lundman [48, 49]. Authors first read and re-read all transcripts to become familiarized with the data and context. Condensed meaning units were then developed related to nurses’ experiences regarding management of patients with DKA. This was followed by manual coding in the margins and synthesizing and grouping of data in relatively exhaustive categories through memos. All authors participated in the data analysis process. In instances of discrepancies in forming codes, categories, discussions were conducted to reach a consensus. Table 1 illustrates an example of how meaning units from transcripts were extrapolated to codes and categories.

**Table 1:** Example of extrapolation of categories from qualitative data, illustrating how meaning units were condensed to codes and generalized to categories.

Meaning unit	Condensed meaning unit	Code	Sub-Category	categories	Theme
*“Until the patient relatives’ to bring the required equipment/drugs involves a long process causing delaying to initiate the management*. *Once the doctor writes the prescription*, *the relative has to go to the pharmacy to have obtain the price of equipment then he/she have to go to the counter to pay*, *then back to the pharmacy to collect the equipment/drugs*. *Those places are not that when he goes he is alone*, *there are other patient relatives’ who also come*. *This means that by the time they reach you*, *a considerable amount of time*, *possibly two hours*, *has already passed*.*”* (Participant no. 6)	Long process of obtaining drugs/equipment at the emergency department consume a considerable amount of time.	Time consuming process	Logistics in Emergency Care	Organization level barriers	Barriers to DKA management
*“…*. *truly*, *there is no established efficient system …*. *for a patient to receive services first and then pay later*, *they [hospital management] want the patient to pay before receiving the service and that’s the challenge we face*.*”* (Participant no. 3)	There is no efficient system for the patient to receive the services first before payment.	Payment challenges			
*You can get insulin*, *but there is no insulin syringe*, *you find yourself stuck because*, *trying to calculate the dose needed from what you had to draw into a regular syringe it’s very difficult*.*”* (Participant no.2)	When Insulin is available, but no insulin syringe. Calculating insulin dose with a regular syringe is challenging.	Insulin-Syringe Availability Challenge	Availability and accessibility of medical devices and supplies		

### 2.7 Methodological considerations

The utilization of approaches such as credibility, transferability, dependability, and conformability guarantees trustworthiness in qualitative studies [50]. To maintain the credibility in the current study all authors discussed together and the concept with the greatest potential for expressing their point of views were picked [51]. To ensure uniformity, the interview guide was used, and two researchers (IMK and KIM), with a third checker (JSA) experienced in qualitative study designs, read and analyzed all transcripts [51]. The study’s conformability was established by using quotes from participants and accomplishing methods of credibility, transferability, and dependability [40].

### 2.8 Ethical considerations

This study was approved by the Research Ethics Board of Muhimbili University of Health and Allied Sciences with Ref. no DA 282/298/01.C/1741. Permission to conduct the study was granted by Temeke RRH with Ref. No TRRH/RSC/9/9/02/8 and Mwananyamala RRH with Ref. no MA. 239/240/01/102. Therefore, before beginning an interview, all participants were informed with comprehensive explanations of the study’s purpose, procedure, risk and benefits both in verbal and written form. They were informed that their participation was voluntary, confidentiality of their information by not using their personal identification information. After ensuring that all their questions were satisfactorily answered and they fully understood the study, participants were asked to sign the provided written consent form.

## 3. Findings

### 3.1 Participant demographic characteristics

A total of 12 nurses participated in this study, with eight of them being males. Most of them (eight) had one year working experience at Emergency Medical Department, nine were holders of diploma in nursing (assistant nursing officers). Table 2 summarizes participants’ demographic characteristics.

**Table 2:** Demographic characteristics of study participants (N = 12).

Participant No.	Sex	Age	Nursing Category	Level of Education	Experiences working at EMD
1.	Male	26	Nursing Officer	Bachelor of Science in Nursing	1 Year and 2 Months
2.	Female	32	Assistant Nursing Officer	Diploma in Nursing	1 Year and 6 Months
3.	Male	36	Assistant Nursing Officer	Diploma in Nursing	1 Year
4.	Male	29	Nursing Officer	Bachelor of Science in Nursing	2 Years and 6 Months
5.	Female	36	Assistant Nursing officer	Diploma in Nursing	1 Year
6.	Male	26	Assistant Nursing Officer	Diploma in Nursing	2 Years
7.	Male	24	Assistant Nursing Officer	Diploma in Nursing	1 Year and 4 Months
8.	Male	33	Assistant Nursing Officer	Diploma in Nursing	1 Year
9.	Female	28	Nursing Officer	Bachelor of Science in Nursing	9 Months
10.	Male	23	Assistant Nursing Officer	Diploma in Nursing	1 Year
11.	Male	23	Assistant Nursing Officer	Diploma in Nursing	1 Year and Two Months
12.	Female	35	Assistant Nursing Officer	Diploma in Nursing	3 Years and 4 Months

### 3.2 Themes

Two major themes emerged from the interview, namely: (1) Facilitators of DKA management and (2) Barriers to DKA management. The two themes, identified and organized to corresponding sub-theme based on socio-ecological model into; individual level barriers, Interpersonal level barriers, organizational level barriers.

#### 3.2.1 Facilitators of DKA management

Facilitators of DKA management referred to all factors that influenced nurses to effectively manage patient with DKA at the emergency department. Therefore, under this theme three sub- categories were identified; Nurses knowledge of DKA, the availability of DKA management protocol and nurses’ skillset to enhance DKA management.

*a) Nurses’ general knowledge of DKA*. General knowledge and understanding of DKA was revealed as an important factor in managing patients with DKA. Nurses identified and discussed different clinical presentations and risk factors of patient with DKA during nursing assessment when patients arrived at EMD, as demonstrated by a male assistant nursing officer and supported by male nursing officer:

*“That is, they [DKA patients] often present with symptoms such as vomiting, general body weakness, feelings of thirst, decreased urine output, and sometimes an unconscious state.* (Participant No. 6)

*“The first risk factor for someone to develop DKA is having diabetes*, *the second (…*.*) is a patient who has diabetes but is unaware of it*, *and third is other illnesses*, *such as any infection*, *which can precipitate hyperglycemia*, *eventually leading to DKA*.*”* (Participant No. 1)

Overall, the most commonly reported DKA diagnostic criteria was the presence of ketones in the urine and high blood glucose level. Rarely did nurses mention blood pH and bicarbonate as part of DKA Diagnostic criteria, as reported by a male nursing officer:

*“First, with a DKA patient, you’ll typically find a high RBG, which is one of the criteria. Additionally, you should also check the blood’s pH, which should be around 7.3. You’ll also examine the urine for ketones and check ABG gases, such as bicarbonate acid levels, in the blood.”* (Participant No. 4)

*b) The availability of DKA management protocol*. This subtheme highlights the presence of general DKA management protocol that available in Emergency department. Adherence to established DKA protocols is crucial for enhancing the quality of care provided to patients with DKA in the emergency department. Nurses emphasized the importance of following these protocols to ensure consistent and effective management. A male nursing officer with two years of experiences highlighted the accessibility and utilization of these protocols:

*“In our emergency department, in each resuscitation room has emergency protocols posted including DKA protocol. So, if a patient is suspected to have DKA, we refer the protocol to see how to initiate management.”* (Participant no. 4)

The specific requirements of the DKA management protocol, particularly during insulin therapy, were discussed by another nurse. That, with the available management protocol helps timely tracing the response of insulin therapy.

*“Anh, the available DKA management protocol wants to check the RBG level after every 15 minutes, during insulin therapy, although we usually stay with the patient for up to three hours.”* (Participant no. 10)

*c) Nurses’ skillset to enhance DKA management*. Nurses discussed the skills they possessed and shared their experiences on what they do when they receive a diabetic patient suspected to have DKA. Recognizing DKA as a life-threatening condition was crucial to them for prompt and effective intervention. A female nurse officer described her initial assessment process:

*“…… if the patient is already known to have diabetes, and I suspect patient might have DKA, the first thing I do is check their RBG. Once I’ve checked the RBG and its high, I also check their urine to confirm whether this patient has DKA or not”* (Participant no. 9)*Top of Form*

The ability to recognize DKA promptly when conducting assessment and act swiftly was further discussed by a male nurse officer, highlighting the critical nature of timely interventions:

*“…. DKA is an emergency condition for us, therefore when I receive DKA patient, I first check vital signs including RGB. Then I insert the double IV lines on the right and left sides of the arm.”* (Participant no. 1)

#### 3.2.2 Barriers to DKA management

Barriers to DKA management refers to factors that affect nurses when managing DKA patient at the emergency department. Therefore, under this theme eight sub-categories were identified; limited training on emergency care, lack of autonomy, decisions disagreement, delayed electrolyte results, scare of medical resources, shortage of nursing staffs, logistics in emergency care, and lack of specific-nursing management guideline.

*Individual level barriers to DKA nursing management*. ***a) Limited training on emergency care*.** Nurses reported either limited or lack of specific training on DKA management in their previous trainings. They perceived this as one of the reasons for not being able to effectively manage patients with DKA, as reported by a female nursing officer:

*“… . There should be training available because we have never been trained on these DKA issues. You know, being trained is different from just reading about it in Classroom.”* (Participant no. 9)

Nurses who at least attended some training which included DKA perceived themselves to have some advantage regarding DKA management, as stated by a female assistant nursing officer:

*“At least, I am familiar with DKA because I attended a short course in ICU care, and we touched on DKA. But there are others nurses who may not know anything about DKA. All they know is to administer fluids without understanding the why and how to them [Nurses] it’s a considerable challenge”* (Participant no. 2)

***b) Lack of autonomy*.** Several nurses reported that, they were dependent on doctors’ decisions to manage a patient with DKA specifically when to initiate insulin infusion. They attributed dependency on doctors to lack of autonomy, as stated by a male assistant nursing officer;

*“You want to do something, but you can’t just do it; you have to wait for the doctor to come and say … administer this amount of insulin….give this amount of fluid.”* (Participant no. 11)

*“So*, *when I have measure patient RBG*, *I will inform the doctor*, *‘doctor*. *patient RGB is still high what do we do*?*’ and the doctor is the one who will make decision to either increase the fluid or start insulin therapy*.*”* (Participant no. 3)

*Interpersonal level barriers to DKA nursing management*. ***c) Decisions disagreement*.** When managing patients with DKA, nurses talked about decision disagreement among nurses and doctors hindered their autonomy to decide on the course of action during management of DKA patient at the emergency department. The male assistant nursing officer stated:

*“You can make certain decisions based on your assessment of the patient and management protocol, but the doctor may not agree. Despite the time you’ve spent with the patient, observed patient condition closely, but the doctor suggests something different from the management protocol. It becomes a significant challenge to decide on a course of action. In this situation, you are advocating for your perspective, and the doctor is advocating for his perspective.”* (Participant no. 10)

***d) Delayed electrolyte laboratory results*.** The waiting times for laboratory results in emergency department were also highlighted. Where Nurses describe a significant inefficiencies and delays electrolyte results thus impacting patient care, as reported by male assistant officer;

*“Ah, we usually take blood sample while doing cannulation and send sample directly to the laboratory. So, while continuing with the management, we wait for at least two hours to get the initial lab results that come while the patient is still in emergency department. Other results including electrolyte results, which tend to take longer, reach the patient in the ward.”* (Participant No. 10)

Where, nurses often find it necessary to personally intervene the process of obtaining lab investigations to shorten the waiting time of results when caring DKA patients. as reported by a male nursing officer;

*“…. At times, the hospital laboratory can get congested. In such situations, I personally go to the lab and ask for urgently needed results. By being there [in the laboratory], I can get the receipt of the test results in short time which it might take a bit longer by not being there….”* (Participant no. 4)

*Organizational level barriers to DKA nursing management*. ***e) Availability and accessibility of medical devices and supplies*.** Participants in this study reported instances of ineffective management DKA patients at EMD caused by challenges related to the availability of the point of care devices. In such cases samples had to be sent to the hospital laboratory for investigations, including electrolytes tests, which often resulted in unusual delays in managing DKA patients, as reported by a male nursing officer:

*“We don’t have our own Point of Care devices here in the emergency room to measure and monitor these electrolytes, so we have to send blood sample to the lab.”* (Participant no. 4)

One nurse shared his experience of managing a DKA patient who collapsed due to delays in investigation results as they just waited without knowing the patient’s blood levels of potassium:

*“The relative made the electrolyte investigation payment early, but investigation results delayed. So, initiating insulin became a challenge, we just had to wait, since we didn’t know the patient level of potassium. In the end, we supported the patient with IV fluids only, but the patient collapsed."* (Participants no. 1)

The other challenge nurses experienced was the availability of insulin and insulin syringe which was reported to be limited, making it difficult to manage patients with DKA at the emergency department as reported by a female assistant nursing officers and emphasized by male assistant nursing officer:

*“You could get insulin, but if there’s no insulin syringe, you might find yourself stuck because, calculating the dose needed from what you had to draw using the regular syringe it’s very difficult”* (Participant no 2)

*"……*. *challenges we encounter is the limited availability of medical supplies*, *and this is aggravated by the reliance on patients’ relatives to bring in some of the necessary supplies from hospital pharmacy……*, *when we decide to administer insulin to a patient*, *obtaining the required insulin from the patient’s relatives pose difficulties*. *This situation potentially lead to the patient receiving a suboptimal insulin dose…”* (Participant no. 10)

However, a situation was different from what was initially experienced when medical devices and supplies were available at the emergency department which helped in effective patient care as reported by the emergency nurse in charge:

*“…. initially, we had access to those resources like the ‘I start’ kits, we had everything available, which contributed to effective patient care, and potassium chloride was readily available. I think the availability of point of care devices and medications certainly made a difference in patient care”* (Participant no. 2)

***f) Shortage on nursing staff*.** Inadequate staffing was reported to hinder provision of effective care to DKA patients, as nurses were overwhelmed with work, specifically during the night shifts when only one nurse worked in the resuscitation room. The one nurse was responsible for everything such as taking admitted patients to wards and for investigations, as stated by a female nursing officer:

*“In one night, you might attend to around 20 patients per shift, and that’s challenging, especially considering that you don’t have a runner to assist you. You are responsible for everything, from taking the patients to their admitted wards, to getting them to undergo tests like CT scans, X-rays, and ultrasounds it’s all on you, so it is demanding.* (Participant no. 9)

Due to shortage of staff, particularly during night shifts, medical attendants were occasionally allocated to the resuscitation room to manage patients, even though their job descriptions did not permit such responsibilities, as reported by an in-charge nurse.

*“…You may find that in the three resuscitation rooms, I need at least three trained nurses, meaning one trained nurse per resuscitation room. But when you allocate three nurses, including two trained nurses and one medical attendant, you still haven’t accomplished much because they don’t understand DKA or even diabetes; they [Medical attendant] know their basic duties…”* (Participant no. 2)

The importance of adequate number of staff was mentioned as an important factor that would help in proper management of DKA patients as mentioned a male assistant nursing officer

*“If we could have enough number of staff particularly trained nurses with good team work will help to adhere to DKA management protocol”* (Participant no. 7)

***g) Logistics in emergency care*.** Management of DKA was similarly reported to be affected by logistics involved in the acquisition of medical equipment and supplies such as medication, IV fluids and laboratory investigations. Nurses reported the process to be time and energy consuming and eventually delaying provision of timely care to DKA patients as reported by a male assistant nursing officer:

*“Until the patient relatives’ to bring the required equipment/drugs involves a long process causing delaying to initiate the management. Once the doctor writes the prescription, the relative has to go to the pharmacy to have obtain the price of equipment then he have to go to the counter to pay, then back to the pharmacy to collect the equipment/drugs. Those places are not that when he goes he is alone, there are other patient relatives’ who also come. This means that by the time they reach you, a considerable amount of time, possibly two hours, has already passed.”* (Participant no. 6)

Patients were expected to pay for services before they received treatment, which made it difficult for patients who were struggling financially to receive treatment. This was another difficulty faced by nurses when managing patients with DKA: the cost of care, which some relatives could not afford, as verbalized by a male nurse officer and assistant nursing officers:

*“Many patients don’t pay for ABG analysis test, so we start with what they can pay for.”* (Participant no. 1)

*“…*. *truly*, *there is no established efficient system …*. *for a patient to receive services first and then pay later …*. *they [hospital management] want the patient to pay before receiving the service and that’s the challenge we face*.*”* (Participant no. 3)

Sometimes nurses got themselves into trouble when they decided to provide services to patients with serious needs who had not yet paid for services they needed.

*“I might put a guarantee there where I request things like ‘I need normal saline’ Later, when you go to tell the relative to pay for it, they might say they don’t have money. I will have to be held responsible for it. We usually provide a guarantee just to get the service quickly. But sometimes you may find yourself being charged for these medications that you’ve given to the patient.”* (Participant no. 5)

***h) Lack of specific-nursing management guideline*.** This subtheme refers to the absence of detailed and specific guidelines tailored exclusively for nursing management of DKA. Thus the available DKA management guidelines did not specify what nurses and other healthcare workers should do, posing a challenge to its implementation. This was reported as one of the barriers in managing DKA patients among nurses as reported by a female assistant nursing officer:

*“I think the protocol appears to be quite comprehensive, where it might seem like it leaves no distinction between doctors and nurses regarding the management steps, when it comes to a DKA management protocol, what does a nurse do?”* (Participant no. 2)

## 4. Discussion

This study investigated perceived factors influencing the management of patients with DKA among nurses at the emergency medical department. Facilitators and barriers to DKA management were identified. In this section, we further contextualize the findings with the available literature and provide suggestions on how these findings can be useful in improving the management of patients with DKA among nurses at EMD.

Foremost, this study identified three key facilitators influencing the effective management of patients with DKA in the emergency department among nurses. Firstly, nurses’ knowledge of DKA, encompassing an understanding of clinical presentations and risk factors, emerged as crucial influencing factor. General understanding of DKA, including identifying precipitating factors like infections enables targeted nursing assessments and interventions. However, there were notable gaps in diagnostic criteria, as blood pH and bicarbonate were infrequently mentioned in DKA diagnostic criteria. The observed gap could be influenced by either lack of on job training among the participants, absence of point of care tests used to detect blood pH and Bicarbonate level or level of nursing education among participants where most of nurses were having diploma level of education. Therefore, this gap emphasizing the need for comprehensive awareness of DKA management among nurses in the emergency department.

The availability to DKA management protocols were highlighted as essential facilitator. Nurses emphasized the presence of readily accessible protocols in resuscitation rooms, providing guidance on initiating DKA management. This is contrary with the available literature where nursers working in emergency department found to use their past experiences in clinical decision making process in emergency situations [52, 53]. The study conducted in the Netherlands discovered characteristics that impact nurses’ adherence to emergency protocols, such as decreased engagement of nurses during protocol creation and a lack of awareness among nurses viewed as hurdles to adherence to the established procedures [54]. Several measures appear to be particularly helpful in promoting clinical guideline adherence, including educational sessions or distribution of educational materials in combination with clinical audit and other actions for improvement [55, 56]. Furthermore, particular implementation tactics such as modification of recommendations to local contexts, usage of implementation action teams and guideline implementation plans, provider education, and team- and technology-based interventions are required to promote use of published guidelines [57, 58].

The skillset of emergency nurses, contributed to the effective management of patients suspected of having DKA. Nurses demonstrated proficiency in prompt glucose level assessments, urine analysis, and the ability to recognize DKA as a life-threatening condition. Rapid nursing actions, such as inserting double intravenous lines upon patient arrival, were emphasized as critical initial steps when caring DKA patients. Similarly, a research that investigated nurses’ experiences with deteriorating ward patients found that assessing vital signs was a crucial measure in verifying and quantifying deteriorating patients [59]. Furthermore, Brysiewicz et al. emphasize the need of clinical skills and prompt interventions by emergency nurses, which coincide with the observed skills required for DKA care [60].

Moreover, the present study identified the barriers to DKA nursing management. Participants discussed the availability and accessibility of medical devices and supplies were reported to hinder the management DKA patients at emergency department. At the point of care, devices including ABG analysis machine and glucometer, and supplies such as insulin and insulin syringes, were reported to be inadequate or lacking at the emergency department. Similarly with Matthews et al., found a shockingly limited availability of critical items needed for DKA management such as insulin and glucometers among low-income and middle income [61]. Literature identified that nurses’ ability to recognize and respond to patient’s deterioration, reduce adverse events and promote patient safety [62], however Due to inadequate communication, and nurses’ lack of knowledge and understanding of patient deterioration patients in acute care settings get sub optimal treatment, necessitating immediate recommendations for improvement [59, 62, 63]. Study conducted in South Africa also found a critical shortage of medical equipment impacted negatively on nursing care including prolonging stay of patients in the hospital, resulting in prolonged procedures for referral of patient [64].

Shortage of nursing staff plus overwhelming workload reported in the current study as significant barriers to effective management of DKA patients was also found in Canada where heavy workload due to shortage of staff affected nurses’ ability to provide optimal care [30]. Similarly, in Australia and Botswana, nurses perceived a shortage of staffing and low skills mix shift allocations as factors affecting care and causing nurses’ roles to be confused at the EMD [30, 65]. Another study in Tanzania found Nurses and physicians at a regional hospitals were unable to give optimal treatment to critically sick patients due to shortage of critical care prepared personnel [66].This shows how common this problem is in both low- and high-income countries. The importance of an adequate number of trained staff mentioned by participants from this study aligns with the recommendation from Wolf et al., who concluded that both absolute numbers of staff, as well as skill and experience mix, should be considered to provide staffing levels that promote optimal patient outcomes at the EMD [29]. Therefore, adequate Registered Nurses staffing in the Emergency Medical Department relates with improving the missed DKA care, better patient ratings of their care experiences, ensuring safe patient care, and ultimately preventing work overload among nursing staff [67–69].

Furthermore, our study highlighted controversial findings to ISPAD Consensus Guidelines DKA management recommendations where nurses reported limited exposure to DKA management training, scarce medical resources (point of care tests, sometimes insulin), and delayed laboratory investigation as barriers in managing DKA patients at EMD [24]. This discrepancy needs to be addressed to effectively provide care to patients with DKA at emergency department.

Despite the vital roles of emergency nurses in early identification of life threatening conditions, timely crucial intervention and continued proper management [70], in the present study, nurses reported a lack of training regarding managing DKA. Those who attended DKA training reported covering only basic emergency training. A survey of ten hospitals in Tanzania found that 80% of them had no staff trained in adult triage or critical care [71]. Aziz et al. explored the overall emergency nurses’ educational needs and found that nurses claimed training in clinical skills, drugs, sera, disease diagnosis, nursing care, documentation, and patient safety management as the main educational need. Similarly, some of participants claimed there was no preclinical education for the nurses working in emergency department [36]. In another study conducted in Saudi Arabia and Egypt, lack of knowledge and training limited nurses’ ability to recognize DKA manifestation and adherence to the management of DKA effectively [32, 33]. Therefore, addressing the gaps in DKA management training is crucial for enhancing nurses’ preparedness and effectiveness in handling these cases at the emergency medical department to ensure effective initial management of these patient before the transfer to the ICU.

Participants described the challenges related to logistics in acquiring equipment and supplies at the emergency medical department as a time-consuming process resulting in delays in treatment initiation among patients with DKA, ultimately increasing the time of stay at an Emergency Medical Department. These findings are congruent with Mashao who reported prolonged length of stay at EMD during the output phase the decision phase while [72] Van, et al reported that delayed treatment at the EMD was associated with a lack of resources, resulting in a disturbance in patient flow at EMD [73].

Nurses also questioned about their distinct roles in DKA management and the availability of the DKA management protocol, where they reported needing to be more focused on nursing roles when caring for patients with DKA. This posed a challenge for nurses over the extent of their involvement in managing patients with DKA. They recommended improving the specific nursing guidelines essential for promoting a standardized and collaborative approach to DKA management in the EMD. Furthermore, nurses highlighted that effective utilization of DKA management guideline was negatively impacted with decision disagreement and lack of autonomy with other health professionals in the emergency department. An elaborative DKA management protocol and clearly stipulated roles of nurses with regard to managing of patients with DKA at emergency department would be very helpful to address this challenge.

### 4.1 Limitation

Our study had some limitations worth mentioning. First, there may be potential for desirability bias in the responses when addressing questions of decision making and influencing factors. However, through asking participants to describe valid examples of situation personally experienced, desirability bias was minimized. Second, participants representativeness where the selected RRHs may not fully represent the experience and practices of nurses working in High level health facilities (eg. National hospital, zonal hospitals) hence limit the applicability of the findings.

## 5. Conclusion

The findings of the current study highlight facilitators and barriers to DKA management among nurses, underscoring the importance of addressing the barriers by making available specific nursing DKA management guidelines, coupled with adequate training and resource allocation to enhance the capacity of emergency nurses to provide optimal care to patients with DKA. Moreover, the findings suggest the need to consolidate facilitators of DKA management.

## Supporting information

S1 File (PDF)

## Abbreviations

EMD: Emergency Medical Department
DKA: Diabetic Ketoacidosis
DM: Diabetes Mellitus
ICU: Intensive care Unity
RRH(s): Regional Referral Hospital(s)
