# Supplementary material for: Understanding the factors influencing nurses in managing patients with diabetic ketoacidosis in the emergency departments of referral hospitals in Dar es Salaam, Tanzania: A descriptive qualitative study
Source: PLoS One. 2024 Nov 12;19(11):e0310414. doi: 10.1371/journal.pone.0310414 (PMC11556755; doi:10.1371/journal.pone.0310414)
Supplement: S1 File — (PDF) [file pone.0310414.s001.PDF]

Understanding the Factors Influencing Nurses in Managing Patients with Diabetic Ketoacidosis in the emergency departments of Referral hospitals in Dar es Salaam, Tanzania: A Descriptive Qualitative study.

Demographic characteristics of study participants (N=12)

Participant No.	Sex	Age	Professional title	Level of Education	Experiences working at EMD
1.	Male	26	NO	Bachelor of Science in Nursing	1 Year and 2 Months
2.	Female	32	ANO	Diploma in Nursing	1 Year and 6 Months
3.	Male	36	ANO	Diploma in Nursing	1 Year
4.	Male	29	NO	Bachelor of Science in Nursing	2 Years and 6 Months
5.	Female	36	ANO	Diploma in Nursing	1 Year
6.	Male	26	ANO	Diploma in Nursing	2 Years
7.	Male	24	ANO	Diploma in Nursing	1 Year and 4 Months
8.	Male	33	ANO	Diploma in Nursing	1 Year
9.	Female	28	NO	Bachelor of Science in Nursing	9 Months
10.	Male	23	ANO	Diploma in Nursing	1 Year
11.	Male	23	ANO	Diploma in Nursing	1 Year and Two Months
12.	Female	35	ANO	Diploma in Nursing	3 Years and 4 Months

NO: Nursing Officer, ANO: Assistant Nursing Officer

<u>EXCERPTS OF THE TRANSCRIPTS</u>

1.0 Facilitators of DKA management

1.1 Nurses' general knowledge of DKA

That is, they [DKA patients] often present with symptoms such as vomiting, general body weakness, feelings of thirst, decreased urine output, and sometimes an unconscious state. (Participant No. 6)

"The first risk factor for someone to develop DKA is having diabetes, the second (....) is a patient who has diabetes but is unaware of it, and third is other illnesses, such as any infection, which can precipitate hyperglycemia, eventually leading to DKA." (Participant No. 1)

"First, with a DKA patient, you'll typically find a high RBG, which is one of the criteria. Additionally, you should also check the blood's pH, which should be around 7.3. You'll also examine the urine for ketones and check ABG gases, such as bicarbonate acid levels, in the blood." (Participant No. 4)

1.2 The availability of DKA management protocol

"In our emergency department, in each resuscitation room has emergency protocols posted including DKA protocol. So, if a patient is suspected to have DKA, we refer the protocol to see how to initiate management." (Participant no. 4)

"Anh, the available DKA management protocol wants to check the RBG level after every 15 minutes, during insulin therapy, although we usually stay with the patient for up to three hours." (Participant no. 10)

1.3 Nurses' skillset to enhance DKA management.

"…… if the patient is already known to have diabetes, and I suspect patient might have DKA, the first thing I do is check their RBG. Once I've checked the RBG and its high, I also check their urine to confirm whether this patient has DKA or not" (Participant no. 9)

".... DKA is an emergency condition for us, therefore when I receive DKA patient, I first check vital signs including RGB. Then I insert the double IV lines on the right and left sides of the arm." (Participant no. 1)

2.0 Barriers to DKA management

2.1 Limited Training on emergency care

".... There should be training available because we have never been trained on these DKA issues. You know, being trained is different from just reading about it in Classroom." (Participant no. 9)

"At least, I am familiar with DKA because I attended a short course in ICU care, and we touched on DKA. But there are others nurses who may not know anything about DKA. All they know is to administer fluids without understanding the why and how to them [Nurses] it's a considerable challenge" (Participant no. 2)

2.2 Lack of autonomy

"You want to do something, but you can't just do it; you have to wait for the doctor to come and say '... administer this amount of insulin.... give this amount of fluid'." (Participant no. 11)

"So, when I have measure patient RBG, I will inform the doctor, 'doctor. patient RGB is still high what do we do?' and the doctor is the one who will make decision to either increase the fluid or start insulin therapy." (Participant no. 3)

2.3 Decisions disagreement

"You can make certain decisions based on your assessment of the patient and management protocol, but the doctor may not agree. Despite the time you've spent with the patient, observed patient condition closely, but the doctor suggests something different from the management protocol. It becomes a significant challenge to decide on a course of action. In this situation, you are advocating for your perspective, and the doctor is advocating for his perspective." (Participant no. 10)

2.4 Delayed electrolyte laboratory results

"Ah, we usually take blood sample while doing cannulation and send sample directly to the laboratory. So, while continuing with the management, we wait for at least two hours to get the initial lab results that come while the patient is still in emergency department. Other results including electrolyte results, which tend to take longer, reach the patient in the ward." (Participant No. 10)

".... At times, the hospital laboratory can get congested. In such situations, I personally go to the lab and ask for urgently needed results. By being there [in the laboratory], I can get the receipt of the test results in short time which it might take a bit longer by not being there...." (Participant no. 4)

2.5 Availability and accessibility of medical devices and supplies

"We don't have our own Point of Care devices here in the emergency room to measure and monitor these electrolytes, so we have to send blood sample to the lab." (Participant no. 4)

"The relative made the electrolyte investigation payment early, but investigation results delayed. So, initiating insulin became a challenge, we just had to wait, since we didn't know the patient level of potassium. In the end, we supported the patient with IV fluids only, but the patient collapsed." (Participants no. 1)

"You could get insulin, but if there's no insulin syringe, you might find yourself stuck because, calculating the dose needed from what you had to draw using the regular syringe it's very difficult" (Participant no 2)

"……. challenges we encounter is the limited availability of medical supplies, and this is aggravated by the reliance on patients' relatives to bring in some of the necessary supplies from hospital pharmacy……, when we decide to administer insulin to a patient, obtaining the required insulin from the patient's relatives pose difficulties. This situation potentially lead to the patient receiving a suboptimal insulin dose…" (Participant no. 10)

".... initially, we had access to those resources like the 'I start' kits, we had everything available, which contributed to effective patient care, and potassium chloride was readily available. I think the availability of point of care devices and medications certainly made a difference in patient care" (Participant no. 2)

2.6 Shortage on Nursing Staff

"In one night, you might attend to around 20 patients per shift, and that's challenging, especially considering that you don't have a runner to assist you. You are responsible for everything, from taking the patients to their admitted wards, to getting them to undergo tests like CT scans, X-rays, and ultrasounds it's all on you, so it is demanding. (Participant no. 9)

"…You may find that in the three resuscitation rooms, I need at least three trained nurses, meaning one trained nurse per resuscitation room. But when you allocate three nurses, including two trained nurses and one medical attendant, you still haven't accomplished much because they don't understand DKA or even diabetes; they [Medical attendant] know their basic duties…" (Participant no. 2)

"If we could have enough number of staff particularly trained nurses with good team work will help to adhere to DKA management protocol" (Participant no. 7)

2.7 Logistics in Emergency Care

"Until the patient relatives' to bring the required equipment/drugs involves a long process causing delaying to initiate the management. Once the doctor writes the prescription, the relative has to go to the pharmacy to have obtain the price of equipment then he has to go to the counter to pay, then back to the pharmacy to collect the equipment/drugs. Those places are not that when he goes, he is alone, there are other patient relatives' who also come. This means that by the time they reach you, a considerable amount of time, possibly two hours, has already passed." (Participant no. 6).

"Many patients don't pay for ABG analysis test, so we start with what they can pay for." (Participant no. 1)

".... truly, there is no established efficient system .... for a patient to receive services first and then pay later .... they [hospital management] want the patient to pay before receiving the service and that's the challenge we face." (Participant no. 3)

"I might put a guarantee there where I request things like 'I need normal saline' Later, when you go to tell the relative to pay for it, they might say they don't have money. I will have to be held responsible for it. We usually provide a guarantee just to get the service quickly. But sometimes you may find yourself being charged for these medications that you've given to the patient." (Participant no. 5)

2.8 ***Lack of specific-nursing management guideline***

"I think the protocol appears to be quite comprehensive, where it might seem like it leaves no distinction between doctors and nurses regarding the management steps, when it comes to a DKA management protocol, what does a nurse do?" (Participant no. 2)
